# Data protection, interoperability and governance assessment tool: results from a proof-of-concept survey

**DOI:** 10.3389/fdgth.2025.1685774

**Published:** 2025-10-27

**Authors:** Concetta Tania Di Iorio, Elettra Ronchi, Ivan Pristas, Tamara Buble, Nicholas Nicholson, Fabrizio Carinci

**Affiliations:** ^1^ Serectrix Srls, Pescara, Italy; ^2^ Sciences Po, School of Public Affairs, Paris, France; ^3^ Croatian Institute of Public Health, Zagreb, Croatia; ^4^ European Commission, Joint Research Centre, Ispra, Italy; ^5^ Unicamillus International Medical University, Rome, Italy

**Keywords:** data protection, data governance, interoperability, European Health Data Space, preparedness, disease registries, EU health information systems

## Abstract

**Background:**

The Collaborative Health Information European Framework (CHIEF) supports consistent monitoring of quality of care and outcomes, through a cohesive information infrastructure aligned with legal and ethical standards, to ensure preparedness to the European Health Data Space (EHDS). We aimed to define, develop and apply a practical solution to help data controllers and data holders navigating the increasingly complex and rapidly evolving legal conditions for health data governance.

**Methods:**

We designed and applied a modular questionnaire to enable Data Protection, Interoperability and Governance Assessment (DIGA). The tool combines quantitative and qualitative analysis to measure the level of institutional compliance with EU data protection laws, governance standards and the EHDS Regulation. The instrument has been designed to enhance its usability and flexible implementation, allowing users to focus on sections that are considered most relevant for their operational purposes. A test survey was run to test its applicability.

**Results:**

The study demonstrated the tool's effectiveness in capturing real-world practices and help data controllers and data holders in identifying both strengths and critical gaps. Survey results showed that users have already established solid foundations for data protection. Participating centres showed a moderate-to-high capacity to enable the secondary use of health data for both research and public health purposes, reflecting an encouraging level of preparedness for the EHDS Regulation. The user feedback collected alongside the survey confirmed the tool's relevance and usability.

**Conclusions:**

We developed an *ad-hoc* tool to monitor and improve data protection, interoperability and governance, which may represent a strategic resource for disease registries and health information systems. The DIGA tool can support institutional self-assessment, fostering regulatory readiness and generating meaningful insights for the implementation of national and EU-level policies. Further studies are needed to assess the reliability of the tool under different conditions, and refine it accordingly for large-scale implementation. Validation across multiple networks and disease domains within CHIEF will allow strengthening its role in preparation of the EHDS.

## 1 Introduction

The collaborative health information European framework (CHIEF) is an initiative launched in 2022 by European Commission's Directorate-General for Health and Food Safety (DG SANTE) coordinated by the Joint Research Centre. Its primary objective is to provide expert input for the design and implementation of a sustainable EU-wide information system for the periodic collection of harmonised indicators across non-communicable diseases (NCDs).

To address implementation barriers and enablers, CHIEF developed a framework that not only supports consistent monitoring but also ensures alignment with evolving legal and ethical standards. These include compliance with the general data protection regulation (GDPR) (1), adherence to data governance and interoperability best practices and preparedness for the European health data space (EHDS) regulation (2).

This framework is grounded in key EU and international instruments, including the GDPR, the 2016 OECD Council recommendations on health data governance, EHDS Regulation, as well as ENISA guidelines among others. These instruments collectively inform the governance of health information systems (HIS), including disease registries, and guide best practices such as secure data linkage.

Against the backdrop of increasing regulatory complexity and diverging national interpretations of data protection legislation, there is a growing need for practical tools to help data controllers and data holders navigate the evolving legal landscape, without compromising the availability, quality, or utility of health data (3, 4). Although numerous practical and heterogeneous approaches have been developed to provide guidance, the variability of regulatory requirements and implementation contexts shows that a one-size-fits-all solution is not feasible. A compliance toolkit must therefore be both practical and context-sensitive, offering clear direction while accommodating different national and organisational environments.

To meet this need, CHIEF has set out to develop the data protection, interoperability and governance assessment (DIGA) tool, a comprehensive self-assessment instrument that aims to generate quality improvement in data management, as well as reliable information and knowledge for policy-making.

The tool is a comprehensive self-assessment framework designed to help data controllers and data holders of health information systems (HIS)/registries evaluate their level of compliance with EU legislation on data protection and their alignment with best practices in data governance, ethical standards and interoperability, as well as their preparedness to the EHDS regulation.

In particular, the main objective of the DIGA tool is to assess/score the extent to which centres that handle health data in disease registers/HIS adhere to this framework.

Importantly, the DIGA tool is not designed to produce rankings or league tables but to support quality improvement in the management and governance of HIS. By identifying areas for improvement and offering a structured approach to compliance, it enables the adoption of corrective measures that enhance the robustness, interoperability, and legal conformity of health information systems and registries across Europe.

It also provides valuable information for national and EU policymaking through its ability to generate aggregated performance assessments across participating centres; offering key insights for decision-making. This feature becomes increasingly relevant as the number of participating centres using the DIGA tool continues to grow, enhancing its representativeness and policy impact.

## 2 Materials and methods

### 2.1 The data protection, interoperability and governance assessment (DIGA) tool

The development of DIGA within the CHIEF initiative followed a structured process that builds upon the privacy and ethics impact and performance assessment (PEIPA) methodology (5, 6).

First, an extensive literature/scoping review and legal analysis was carried out to identify the privacy, data governance, interoperability and ethics framework for disease registries/health information systems and derive the key elements/factors for analysis; thus, ensuring both conceptual robustness and practical relevance of the questionnaire content (7).

Second, the earlier PEIPA questionnaire (5, 6) was revised, adapted and expanded with new sections on governance, interoperability, GDPR compliance, EHDS readiness, and ethics to thoroughly cover the areas of interest identified in the literature and legal review. The objective was to create a more comprehensive tool for self-assessment while also generating evidence and insights to inform policy-making. The scoring systems, elaborated to support an objective assessment of users' practices, was based on the PEIPA's system (5, 6), though extensively updated and expanded to include scores to new and revised factors relative to data protection, governance, interoperability and preparedness to the EHDS Regulation. The custom-build analysis software was instead maintained in its original format (5, 6). The resulting DIGA questionnaire and scoring system was internally revised by CHIEF Members and further refined based on feedback received.

Finally, the DIGA tool was tested in a proof-of-concept survey to assess its practical feasibility and conceptual robustness

The DIGA tool includes:

• The DIGA Questionnaire, composed of 3 sections, each divided into sub-sections (the factors for analysis) containing a variable number of questions. In total, the questionnaire includes 167 questions (see Supplementary Materials);
• The DIGA scoring system, equipped with a custom-built analysis software capable of performing quali-quantitative analysis of data protection, data governance, interoperability and ethics practices. The scoring tables are available in the Supplementary Materials.

Although the expanded scope of the questionnaire has impacted on its length, it is structured into independent sections. This modular design allows respondents to complete only the sections relevant to their specific context, interests, or operational responsibilities.

The key elements (factors for analysis) of the DIGA tool are grouped in 3 areas: data protection, data governance, and interoperability/preparedness to the EHDS regulation, as described in Table 1.

**Table 1:** Key elements of the DIGA tool.

Section/area	Content/factors
Section/Area 1: Data Protection Requirements	• Legal base for Data Processing: National or EU Legislation • Legal base for Data Processing: Consent • Data subjects’ rights • Accountability • Safeguarding Personal data. This sub−section is further divided into five items: ◦ Security measures for privacy protection ◦ Privacy−by−design and privacy−by−default ◦ Data breach notification and communication ◦ Data protection engineering: anonymisation ◦ Data protection engineering: pseudonymisation • Data linkage
Section/Area 2: Data Governance and Ethics	• Governance framework • Access control and auditing • Data quality and integrity • Training and awareness • Data sharing and collaborations • Openness and transparency • Health research project approval processes • Ethics
Section/Area 3: Interoperability/Preparedness for the EHDS Regulation	• Electronic Health Records (EHRs) content requirements and the European Electronic Health Data Exchange Format • Registries/HIS requirements for interoperability with EHR Systems • Data Documentation, quality and utility • Secondary use of health data

The scoring system applies a standardised coding to yes/no/not applicable (N/A) responses to DIGA items in order to allow a quali-quantitative analysis for all questions and factors included in the questionnaire. In particular:

• A score of 1 is assigned to any conduct that is privacy-protective or compliant with data governance/interoperability/ethics, regardless of whether the response is a yes-no-n/a. Weighted scores are applied where necessary, as specified in the scoring tables (see the scoring tables in the Supplementary Materials);
• A score of zero is assigned to any non-privacy protective or not compliant practice;
• N/A responses to single questions are assigned either a “O” score (most cases) or “1” in selected cases;
• Missing and N/A responses relative to entire sections have been excluded from the calculation of mean, median and total scores.

The custom-built analysis produces descriptive statistics that show a centre's results by factor and by area, compared with the sample's mean, median and the maximum obtainable score. It also displays average and median scores achieved by the whole sample, for each factor and overall, while the variability is examined and graphically described through turnip charts.

Accordingly, results from the quali-quantitative analysis are able to:

• Evaluate the overall level of data protection, data governance, interoperability and ethics in the sample of registries/health information systems involved in the survey;
• Anonymously assess current practices in the handling of sensitive health data in the field of diabetes by scoring the adherence of participating centres to data protection, data governance, interoperability and ethics requirements, bench-marked against the mean and median of the sample, as well as against the best attainable results;
• Evaluate the variability in the implementation of data protection, data governance, interoperability and ethics requirements among participating centres and uncover overly restrictive practices that exceed the provisions laid out by relevant legislation and best practices;
• Identify key areas of concern in the implementation of privacy, data governance and interoperability principles/requirements across participating centres.

### 2.2 Proof-of-concept survey

As a proof of concept, a paper-based version of the DIGA questionnaire was administered to three CHIEF participants who routinely handle large volumes of sensitive data in their capacity as data controllers. These centres operate population-based registries/HIS with national coverage, each serving more than 10% of the target population. They include a national health insurance fund, a national diabetes registry and a national public health information system. They are hereafter referred to as Centre A, B and C. The questionnaire was addressed to data controllers and/or data protection officers and/or chief executive officers responsible for data processing of disease registries/HIS. However, data controllers were advised to consult experts from their IT departments to complete sections that required IT expertise (e.g., security, data breach management, anonymisation and pseudonymisation techniques, data protection engineering, etc.). Involved centres already use other self-assessment tools such as ISO 270001 information security, data quality self-assessment tools, cybersecurity self-assessment, etc.

The main goal of the proof-of-concept exercise was to demonstrate the feasibility of the DIGA questionnaire and scoring system as a useful framework and toolbox for the management of health and social data and for drawing insights for policymaking at local, national and European levels.

Accordingly, the proof-of-concept aimed to:

• Evaluate the suitability of the questionnaire and scoring system as an anonymous self-assessment tool and checklist to be used by data controllers/holders of disease registries/health information systems.
• Assess the tool's effectiveness in helping respondents gauge their level of compliance with privacy/data-protection requirements, interoperability readiness, adherence to data governance best practices and alignment with interoperability and ethical standards as set out by the GDPR, the EHDS Regulation, OECD. guidelines and recommendations and EU ethics rules.
• Improve the ability of the tool to evaluate:
  ◦ The overall aggregate performance of the participating centres in the areas of analysis;
  ◦ Key areas of concerns related to data protection, governance, interoperability, and ethics;
  ◦ The degree of heterogeneity across the sample in terms of implementation and compliance.
• Gather feedback on the complexity, difficulty, feasibility, utility and completeness of the questionnaire to allow a cross-validation of the questionnaire by end-users.

The overall results of the sample were made available to the wider community in a de-identified and/or aggregated format to ensure confidentiality and anonymity of both respondents and Centres involved in the survey.

## 3 Results

The DIGA tool produces two broad categories of results:

• *Centre level results*, describing disease registries/HIS level of compliance with legal requirements and best practices in the implementation of privacy/data protection, data governance, interoperability/preparedness to the EHDS regulation and ethics with regard to data protection/privacy. These results are intended for centres’ self-assessment and to inform quality improvement. Sub-categories include:
  ◦ Performance by Factor: Scaled scores for each factor (based on the sum of scaled item scores within that factor)
  ◦ Performance by Area: Scaled scores for each of the four main areas (based on the sum of factor scores within each area)
  ◦ Centre Profiles: Summary profiles highlighting strengths and weaknesses in each area.
• *Overall sample results*, providing an aggregated view across all participating centres, delivering insights for national and EU-level policymaking. Sub-categories include:
  ◦ Main findings from single questions: overall percentage of YES-NO-N/A responses registered by the whole sample for selected questions;
  ◦ Standardised Comparison of factors Results:
    - Mean and median values achieved by the sample by factors, compared with the maximum attainable results;
  ◦ Standardised Comparison of Areas Results:
    - Mean and median values achieved by the sample by areas, compared with the maximum attainable results.

The overall sample's compliance with privacy/data protection, data governance, interoperability and ethics principles in each factor and by area is further categorised according to an agreed range of scores to simplify the evaluation of performances (see Table 2).

**Table 2:** DIGA scores range.

Score	Range
Excellent	Median value ranges from 90% to 100%
Very Good	Median value ranges from 80% to 89%
Good	Median value ranges from 70% to 79%
Fair	Median value ranges from 50% to 69%
Poor	Median value is equal or below the 49%

### 3.1 Centre level results

Centre-level results are assessed at the factor's level for each centre participating in the survey.

The scores by factor are derived from the standardised sum of scores obtained across individual items (questions) within each factor. They reflect the performance of each centre in specific dimensions within the broader area of reference.

For example, the factor “security measures” is composed of seven questions that aim to assess if security measures include pseudonymisation and encryption of personal data, information security management system, regular testing/assessment and evaluation of the effectiveness of the technical and organisational measures, etc. The standardised sum of scores achieved in the seven questions provides the results for the factor security measures.

Results for this factor were homogeneous. Responses show that the 33.3% of the sample obtained the maximum score for this factor. 33.3% of the sample scored just below the mean of the sample (83%) and in line with the median value of the sample (80%); while the reaming 33.3% scored below both the mean and the median values (70%). Results for this factor highlight a good compliance with data protection requirements for this factor. The range of scores spans from 70% to 100%.

**Figure 1:**
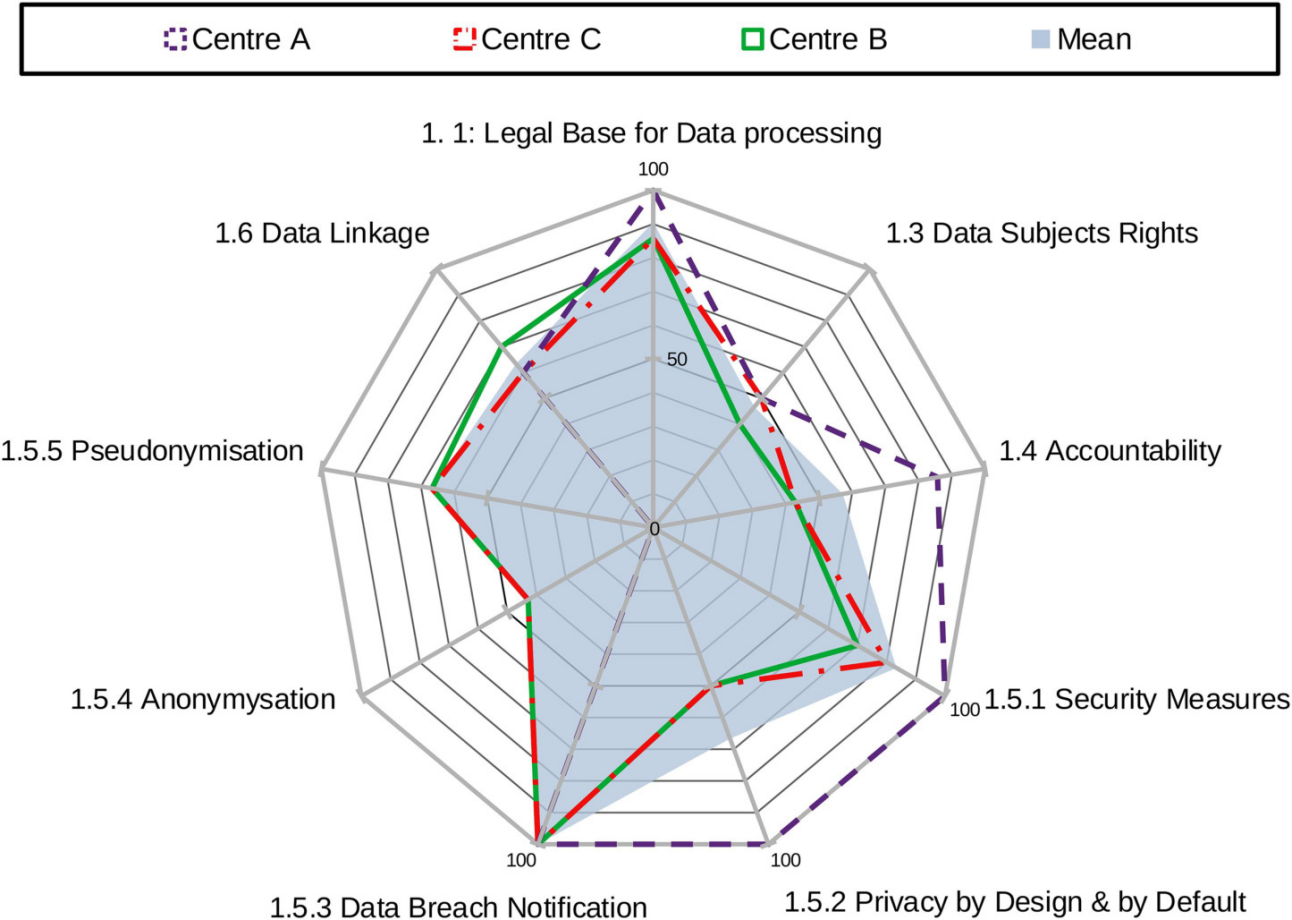
Centre's results by factor in area 1: data protection.

The overall area score for each centre is calculated as the sum of scores across all factors within an area, showing an aggregate measure of compliance in that domain. The standardised sum of scores achieved by each centre across all factors within an area provides the overall result for that area. Figure 1 provides an example of centres' results in area 1: data protection.

**Figure 2:**
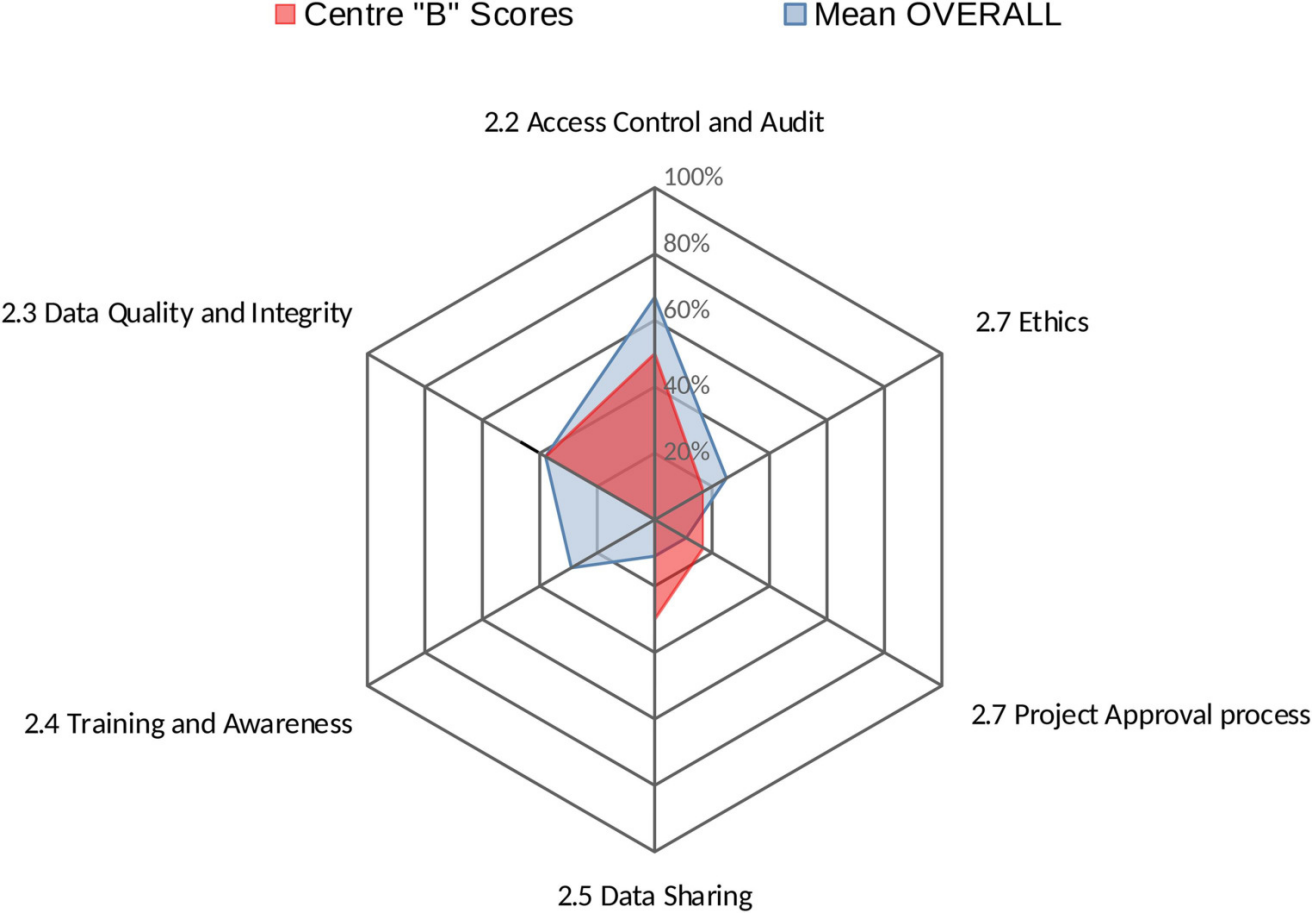
Centre “B” performance in area 2: data governance.

Centres' profiles for each area provide an easy-to-interpret representations of results, summarising in a single graph the performance of a centre across all factors within that area. Figure 2 shows the performance of Centre “A” across all factors within area 2 (Data Governance).

### 3.2 Overall sample results

Main findings from questions within each factor can provide valuable insights into specific areas of interest, aligned with the objectives of the survey. For example, the need to further investigate data linkage, data quality procedures and EHR was dictated by the relevance of these factors for the CHIEF initiative. Nevertheless, similar insights can be obtained for all factors within each area.

Results show that while all centres have the capability to perform data linkage and use unique personal identifiers, standard practices for deleting direct identifiers are not consistently applied; and the methods or processes for de-identification and/or pseudonymisation are not formally documented. Nevertheless, two-thirds of the sample reported using additional protective measures for the treatment of attributes that pose a higher risk of re-identification.

These results unveil the lack of specific practices that could be easily corrected by data controllers or data holders of disease registries/HIS, once known. In this case, applying standard practices to delete direct identifiers and document the pseudonymisation process would increase centres' adherence to legal requirements and best practices.

Similarly, results highlight that data quality assurance standards and protocols are used only in 33.3% of the sample. In those cases, the standards are documented and communicated to relevant staff and responsibilities clearly assigned. However, automated tools or manual processes are commonly used for data validation and verification by two-thirds of the sample.

With regard to EHR, results revealed that two-thirds of the sample use EHRs as routine data source for their registry/HIS. However, a patient summary is typically not available for the same two-thirds. Importantly, the entire sample results indicates that medical images and reports are not included in the EHR system, contrary to what will be required by the EHDS regulation. Finally, two-thirds of the sample do not use the European electronic health data exchange format envisaged in the 2019 Commission recommendation, e.g., Health Level Seven (HL7) clinical document architecture (CDA) release 2, level 3 and level 1 [PDF (3)/A], or CEN/ISO 27269:2021 Health informatics—international patient summary standard.

The investigation of questions within each factor is of utmost important to indicate the non-compliant practices, and direct tailored corrective measures.

The standardised comparison of factor results (the analysis of mean and median values) identifies the areas where the sample obtained “good” to “excellent” scores (see Table 2), i.e., legal base for data processing (median = 86%); security measures (median = 80%); data breach notification (median = 100%); secondary use (median = 75%).

These results indicate that the sample is mostly compliant with relevant legislation and aligns with best practices, recommendations and guidelines for those factors.

Similarly, mean and median values indicate the areas where the sample obtained a “fair” level of performance, suggesting a need for further investigation and/or light corrective measures. Examples include: data subjects rights (median = 50%); privacy by design and by default (median = 50%); access control and audit (median = 57%); requirements for interoperability (median = 50%).

In contrast, factors that show “poor” performance should be regarded as key areas of concern. For example, accountability (median = 43%), anonymisation (median = 43%), data quality and integrity (median = 38%) and EHR Content (median = 36%) achieved a low score in the sample, indicating a likely need for corrective actions.

In the proof-of-concept sample, several factors exhibited high variability in scores (range) indicating inconsistent implementation across centres. These include: privacy by design and by default (range: 50%–100%); access control and audit (range: 50%–100%); data quality and integrity (range: 0%–75%); and training and awareness (0%–86%).

**Figure 3:**
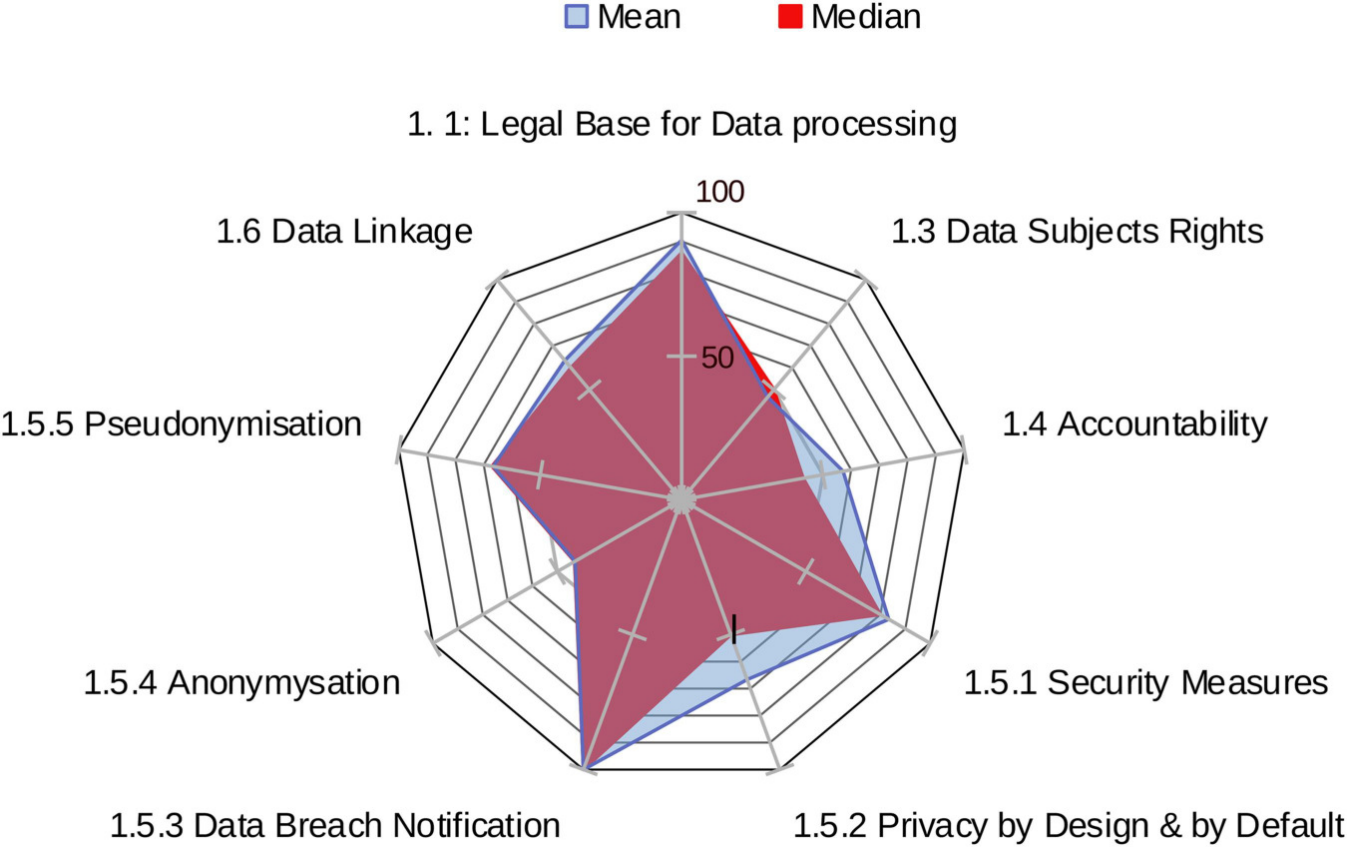
Mean and median values of the sample in data protection.

Figure 3 shows the mean and the median values of the sample against the maximum obtainable scores in the area of data protection by factor. DIGA tool reports also show the range of factors relative to the three areas.

**Figure 4:**
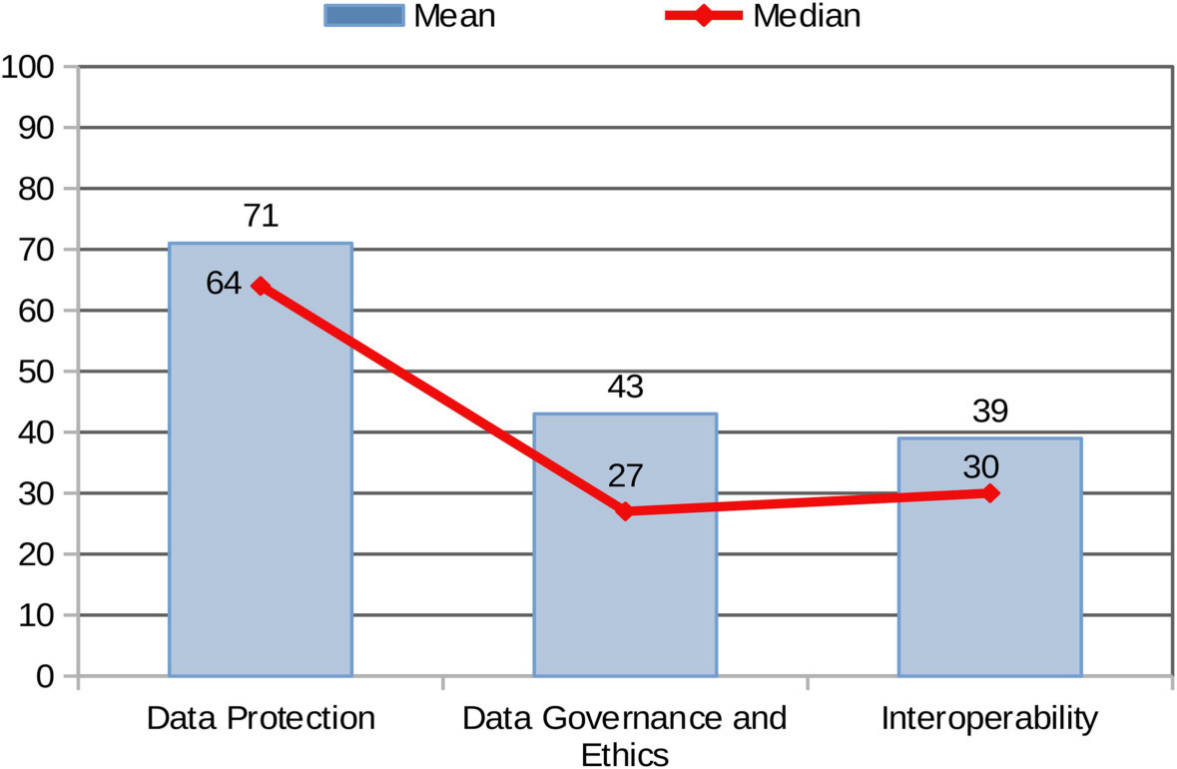
Mean and median of the sample in the 3 areas.

The standardised comparison of results across the three areas (based on mean and median values achieved by the sample by Area) provides a graphical overview of the combined performance of all centres (Figure 4). The sample achieved the highest scores in Area 1: Data Protection, whereas Areas 2 and 3 (Data Governance and Interoperability/EHDS Preparedness to the EHDS Regulation) reported significantly lower mean and median values.

The stronger performance in Area 1 may be attributed to the fact that data protection is legally mandated under the GDPR across the EU, driving more consistent implementation. In contrast, best practices in data governance are currently guided by non-binding recommendations, which may account for the more varied and generally lower performance in Area 2. As for Area 3, the EHDS regulation has only recently entered into force and includes a transitional period extending to 2031. This likely contributes to the lower levels of adherence observed in the Areas: Interoperability and EHDS Preparedness.

### 3.3 Users' feedback results

In parallel with the administration of the DIGA questionnaire, a brief user feedback form was distributed to participating CHIEF centres. The feedback questionnaire, consisted of 10 questions with response options rated as: very low, adequate, and very good.

Results show that the following DIGA tool features were considered “very good”: the anonymity of the individual self-assessment (100% of the sample); the suitability of the DIGA questionnaire to identify key areas of concern (by two-thirds of the sample, while the remaining one-third rated it as “adequate”); the overall utility of the tool as a means for quality improvement (by one-third of respondents, while the remaining two-thirds considered it as “adequate”); and the comprehensiveness of the questionnaire (by two-thirds of the sample, while the remaining one-third evaluated it as “adequate”.

The following features were rated as “adequate” by the 100% of the sample: the suitability of the DIGA questionnaire as a self-assessment tool and checklist; its ability to evaluate the overall level of data protection, data governance, interoperability and ethics; and its suitability to evaluate the heterogeneity of the sample.

Regarding the complexity of the questionnaire, one-third of respondents rated the level of difficulty as “very low”, while the remaining two-thirds considered it adequate. Open-ended feedback also provided useful suggestions for improvement such as reducing the length of questionnaire to support broader utilisation of the tool; limit/avoid multiple response options to increase accuracy; and provide clearer guidance on how to respond when procedures exist in practice but are not formally documented or officially adopted.

## 4 Discussion

High-quality, actionable health information is essential for informed public health decision-making. However, existing data systems across EU Member States often struggle to deliver timely, comprehensive, and interoperable data on NCDs. This is largely due to legal constraints, inconsistent implementation of best practices in data governance and limited interoperability.

Under EU law, the GDPR provides the overarching legal framework for personal and sensitive data processing, including health data (1). It lays down harmonised rules for safeguarding the rights of individuals while outlining the responsibilities of data controllers and processors and of national supervisory authorities. Although health data are considered a “special category” requiring enhanced protection, the GDPR allows specific exemptions [see Art. 9 (2) of the GDPR] enabling processing without consent for reasons of public interest in public health, scientific research, or statistical purposes, provided that appropriate safeguards are in place (e.g., pseudonymisation, anonymisation, and other organisational or technical measures).

Importantly, Art. 9(4) of the GDPR allows Member States (MS) to maintain or introduce additional conditions further expanding or limiting the processing of genetic, biometric or health data. As a result, national level implementation varies significantly. For example, Finland has enacted legislation on the secondary use of health and social data that allows the secondary use of data, without individual consent, for pre-defined purposes (e.g., research, knowledge management, etc.), subject to approval by Findata, a national data intermediary (8).

Conversely, other countries have taken a more restrictive approach, underutilising the exemptions provided by the GDPR, which has impeded an optimal use of data for their national registries and HIS (9) towards a coherent EU information infrastructure (10).

A recent EU-wide assessment of MS rules on health data has highlighted that this legislative and regulatory fragmentation, particularly the uneven implementation of GDPR, remains a major barrier to effective health data use for public health purposes and health systems strengthening (11).

Therefore, a key pre-requisite for building a sustainable HIS is an harmonised implementation of the GDPR, supported by national legislation that enables a broad use of health and health-related data for secondary purposes when in the public interest (e.g., registries/HIS functions), and accompanied by appropriate safeguards (e.g., organisational, technical and security measures) to ensure the protection of data subject rights.

This vision aligns with international best practices. The OECD's advisory panel of experts on health information infrastructure identified in 2015 eight key data governance mechanisms to support privacy-protective data use, aimed at facilitating the secondary use of data for public health, research and statistical purposes (12). Building on these, the 2016 OECD Council recommendation on health data governance (13), calls on countries to develop national data governance frameworks that promote data availability and use, while ensuring privacy protection and data security. These frameworks should also be harmonised across countries to enable multi-country statistical and research initiatives.

In parallel to these international efforts, at the EU level, the 2020 European strategy for data (14) envisaged the creation of EHDS, to unlock the full potential of health data for improved healthcare, research, and policymaking. This goal has since materialised with the adoption of the EHDS regulation in March 2025 (2).

The EHDS regulation introduces specific rules for the health sector aimed at addressing existing legislative gaps related to health information systems and the exchange of electronic health data.

The EHDS regulation establishes a common legal and technical framework for the primary and secondary use of electronic health data across the EU. It mandates two interoperable, cross-border infrastructures: “MyHealth@EU” enabling the primary use of electronic health data across the Union; and “HealthData@EU”) for secondary uses such as research and policy. The regulation also establishes governance mechanisms, interoperability standards, and cybersecurity requirements, all of which must operate in compliance with the GDPR and related legislation, including the NIS2 Directive (15) and the Cybersecurity Act (16).

Despite these advances the complexity of the overlapping legal requirements, combined with diverging national interpretations and practices within the evolving EU legal scenario for health data processing continues to pose challenges for health data use in registries and HIS.

Hence, there is an urgent need for practical tools to support data holders and controllers in navigating this regulatory landscape, ensuring compliance without compromising the availability and quality of information needed for registries and HIS functions.

There are numerous toolkits and guides available designed to help organisations of all sizes achieve compliance with the GDPR, ensuring privacy, security, and accountability in handling personal data. These toolkits are mainly sponsored or published by member states' privacy enforcement authorities/regulators. The CNIL in France has for example developed the GDPR toolkit (17) which, provides a diversified toolbox enabling organisations to dynamically manage and demonstrate their compliance with the regulation: records of processing activities, information statements, data protection impact assessments, transfer frameworks, legal frameworks, certifications or codes of conduct.

Additionally, there are state-of-the-art publications on implementing the GDPR technical requirements, including data encryption, authorisation, and access control, and consent management. Valuable guidance in this regard is provided by the European Data Supervisor (18), EU projects (19) as well as legal consultancy companies.

A recent comprehensive narrative literature review retrieved more than 300 publications on GDPR compliance and implementation guides (3) representing a highly heterogeneous ecosystem of approaches. These findings underscore the necessity of a nuanced understanding of the implementation context in developing a compliance toolkit as there is no one-size-fits-all approach.

Accordingly, the DIGA tool has been developed to evaluate HIS specific contexts and practices (e.g., profile of participating centre) in order to provide tailored guidance on privacy protective uses of health data in the performance of health information system functions (i.e., health monitoring, surveillance, health system performance, public health, health intelligence, etc.). In particular, its scope is twofold:

• a self-assessment tool to generate quality improvement in the management and governance of HIS (including for educational and awareness raising purposes)
• an evaluation tool to generate aggregated performance assessments to inform policy making at both national and EU level, highlighting trends and trajectories in areas of analysis, as well as areas of concern that might need corrective measures.

To the best of our knowledge, available tools do not cover an evaluation of the data governance practices and interoperability in HIS, along with GDPR and EHDS compliance.

The DIGA tool, developed as a core element of CHIEF, aims to address this gap.

The DIGA tool is not aimed to replace or complement the data protection impact assessment (DPIA), which is a legal requirement under the GDPR, but to help data controllers to manage their data in compliance with legal requirements without jeopardising information content for research and public health purposes. In DIGA, the legal compliance evaluation expands to upcoming legislation such as the EHDS regulation, as well as to data governance best practices. Therefore, the DIGA tool could be rather seen as a preliminary compliance check (with regard to the data protection section) that offers tailored solutions to ensure a positive outcome of the DPIA.

The implementation of the tool in the context of the CHIEF initiative provided an opportunity to explore its utility and gather baseline data on practical approaches in the handling of sensitive data across a sample of participating centres.

Among the participating centres, data protection emerged as the best-performing area, an expected outcome given the binding nature of the GDPR across the EU. However, several data protection factors scored lower and warrant further improvement, particularly: data subjects’ rights (median = 50%), privacy by-design and by-default (median = 50%) accountability (median = 43%), and anonymisation (median = 43%).

The implementation of data governance practices showed significant variability, highlighting key areas of concern with regard to: data quality and integrity (median = 38%), training and awareness (median = 0%) and data sharing (median = 30%). These results suggest that while general compliance frameworks are in place, practices around quality assurance, training, and data exchange remain weak, indicating areas where closer alignment with relevant principles, recommendations, and guidelines is needed.

The preparedness for the EHDS regulation scored lowest, likely due to the regulation's recent entry into force and requirements will partially start to take effect from 2027 on. Nonetheless, the findings highlight important areas that need improvement, such as essential EHR content (e.g., availability of patient summaries) and interoperability measures. Addressing these will not only support future compliance with the EHDS but also help overcome existing barriers to the implementation of digital health information systems, whether EHRs systems, registries, or other health platforms.

Interestingly, results indicate a good level of preparedness regarding the secondary use of data in the sample of participating centres, consistent with the objectives of the EHDS regulation and the growing recognition of data reuse as a public good.

While these results offer valuable insights, the small sample size means the findings cannot be considered statistically representative at EU level. Moreover, they cannot be used to infer systematic divergence in data protection and governance practices beyond the analysed centres. Nonetheless, the self-assessment results provide a useful snapshot of current operational practices and suggest key areas where support tools such as DIGA can contribute to ongoing improvements.

A full-scale CHIEF pilot planned for 2025–2026, will involve 17 participating centres and 5 collaborating institutions across Europe. This expanded implementation will allow broader validation of the tool and its capacity to support harmonisation, monitoring, and quality assurance in registries and HIS.

Feedback from participating centres confirmed the tool's suitability for its intended purpose, offering a clear and detailed view of the current practices in participating centres. However, to strengthen its evaluation, future efforts should involve a wider community of users and stakeholders. The upcoming full-scale pilot will expand the user feedback component and explore additional methods such as interviews or focus groups to capture more in-depth perspectives.

## 5 Conclusions

The DIGA questionnaire and scoring system can provide CHIEF with a practical and specific tool that can be used to assess the compliance of disease registries and health information systems with legal requirements and ethical standards in data protection. It also helps identify best practices in the implementation of privacy and data protection, data governance, interoperability, and ethics.

A broad pool of potential users and stakeholders could benefit from using this tool, including data controllers and processors, who could then use the DIGA tool to self-assess their performance as a means of continuous quality improvement and legal/regulatory compliance monitoring.

However, the effectiveness of self-assessment tools depends on the accuracy of self-reported information and the extent to which respondents engage with all relevant sections of the tool. The DIGA tool takes into account these issues, respectively, by advising users to seek complementary expertise for the filling in of the different sections of questionnaire and by ensuring the anonymity of both users' responses and centres' profiles.

The ability of the DIGA tool to generate aggregated performance assessments across participating centres offers valuable insights for EU and national policymaking which will become increasingly relevant with the growing of the sample that uses the DIGA tool.

Therefore, the DIGA tool developed within the CHIEF initiative represents a concrete step forward in supporting EU and national efforts to strengthen legal and ethical compliance in health data use. It emerges as a tool with practical value and strategic resource for disease registries/HIS. It supports institutional self-assessment, strengthens regulatory readiness, and generates meaningful insights to guide both national and EU-level policy.

As the full-scale pilot progresses, the DIGA tool is well positioned to play a pivotal role in the realisation of the EHDS and contribute to a more cohesive, ethical, and data-driven public health ecosystem across Europe.

## Data Availability

The original contributions presented in the study are included in the article/Supplementary Material, further inquiries can be directed to the corresponding author.
