# Supplementary material for: Data protection, interoperability and governance assessment tool: results from a proof-of-concept survey
Source: Front Digit Health. 2025 Oct 27;7:1685774. doi: 10.3389/fdgth.2025.1685774 (PMC12597924; doi:10.3389/fdgth.2025.1685774)
Supplement: Supplementary file 1 [file Datasheet1.pdf]

Supplementary Data

1. DIGA Questionnaire

How to fill in the Questionnaire

The questionnaire is addressed to data controllers and/or data protection officers and/or chief executive officers eventually responsible for data processing of disease registries/HIS.

The questionnaire is composed of four sections (factors), each divided in sub-sections (sub-factors) containing a specific number of questions (items).

Respondents are required to provide YES/NO responses to a series of questions contained in each questionnaire section:

- Section 1 contains 6 sub-sections (total number of questions = 69)
- Section 2 contains 3 sub-sections (total number of questions = 53)
- Section 3 contains 3 sub-sections (total number of questions = 21)
- Section 4 is not divided in sub-sections (total number of questions = 12)

For a total of 155 questions overall.

"N/A" (not applicable) option is also available for cases where single questions or one or more entire sections or sub-sections are not applicable to the respondent.

The "Provide Details" column should be used to explain responses or to provide specific references. It is recommended to provide comments and details in accurate and comprehensive manner in order to allow eventual revisions.

Definitions

Personal data means any information relating to an identified or identifiable natural person ('data subject'); an identifiable natural person is one who can be identified, directly or indirectly, in particular by reference to an identifier such as a name, an identification number, location data, an on-line identifier or to one or more factors specific to the physical, physiological, genetic, mental, economic, cultural or social identity of that natural person (Art. 4 of the GDPR).

Processing means any operation or set of operations which is performed on personal data or on sets of personal data, whether or not by automated means, such as collection, recording, organisation, structuring, storage, adaptation or alteration, retrieval, consultation, use, disclosure by transmission, dissemination or otherwise making available, alignment or combination, restriction, erasure or destruction (Art. 4 of the GDPR).

Pseudonymisation means the processing of personal data in such a manner that the personal data can no longer be attributed to a specific data subject without the use of additional information, provided that such additional information is kept separately and is subject to technical and organisational measures to ensure that the personal data are not attributed to an identified or identifiable natural person. Personal data which have undergone pseudonymisation, which could be attributed to a natural person by the use of additional information should be considered to be information on an identifiable natural person (Art. 4 of the GDPR).

Anonymous data means data which does not relate to an identified or identifiable natural person (‘data subject’) or personal data rendered anonymous in such a manner that the data subject is not or no longer identifiable using any reasonable means. To ascertain whether means are reasonably likely to be used to identify the natural person, account should be taken of all objective factors, such as the costs of and the amount of time required for identification, taking into consideration the available technology at the time of the processing and technological developments (Art. 4 of the GDPR).

Data controller means the natural or legal person, public authority, agency or other body which, alone or jointly with others, determines the purposes and means of the processing of personal data; where the purposes and means of such processing are determined by Union or Member State law, the data controller or the specific criteria for its nomination may be provided for by Union or Member State law (Art. 4 of the GDPR).

Processor means a natural or legal person, public authority, agency or other body which processes personal data on behalf of the data controller (Art. 4 of the GDPR).

Recipient means a natural or legal person, public authority, agency or another body, to which the personal data are disclosed, whether a third party or not (Art. 4 of the GDPR).

Third party means a natural or legal person, public authority, agency or body other than the data subject, data controller, processor and persons who, under the direct authority of the data controller or processor, are authorised to process personal data (Art. 4 of the GDPR).

Consent of the data subject means any freely given, specific, informed and unambiguous indication of the data subject’s wishes by which he or she, by a statement or by a clear affirmative action, signifies agreement to the processing of personal data relating to him or her (Art. 4 of the GDPR).

Personal data breach means a breach of security leading to the accidental or unlawful destruction, loss, alteration, unauthorised disclosure of, or access to, personal data transmitted, stored or otherwise processed (Art. 4 of the GDPR).

Data concerning health means personal data related to the physical or mental health of a natural person, including the provision of health care services, which reveal information about his or her health status (Art. 4 of the GDPR).

Supervisory Authority means an independent public authority which is established by a Member State pursuant to Article 51 of the GDPR (Art. 4 of the GDPR).

Record linkage refers to a merging that brings together identifiable records from two or more sources of data with the object of consolidating facts concerning an individual or an event that are not available in any separate record (Handbook of Vital Statistics Systems and Methods, Vol. 1: Legal, Organizational and Technical Aspects, United Nations Studies in Methods, Glossary, Series F, No. 35, United Nations, New York, 1991.) An example would be linking patient records in a hospital database to any death records for the same persons in a mortality registry in order to identify patients who died following treatment. Deterministic record linkage, often referred to as exact matching, occurs when a unique identifier or set of identifiers is used to merge two or more sources of data. In health linkages, the identifier used is often a unique patient identifying number or UPI. Probabilistic record linkage occurs when a set of possible matches among the data sources to be linked are identified. For example, identifying information such as names, dates of birth, and postal codes, may

be used to assess potential matches. Then statistics are calculated to assign weights describing the likelihood the records match. A combined score represents the probability that the records refer to the same entity. Often there is one threshold above which a pair is considered a match, and another threshold below which it is considered not to be a match. This technique is used when an exact match between records across databases is not possible, or when data capture errors have caused deterministic matches to fail.

Secondary use of data is the processing of personal data for purposes other than those for which the personal data were initially collected [Article 5(1)(b), Articles 6(4) and 89(1) and Recitals 39 and 50 of the GDPR]. The secondary use should be allowed only where the processing is compatible with the purposes for which the personal data were initially collected. In such a case, no legal basis separate from that which allowed the collection of the personal data is required. If the processing is necessary for the performance of a task carried out in the public interest or in the exercise of official authority vested in the controller, Union or Member State law may determine and specify the tasks and purposes for which the further processing should be regarded as compatible and lawful. Further processing for archiving purposes in the public interest, scientific or historical research purposes or statistical purposes should be considered to be compatible lawful processing operations. The legal basis provided by Union or Member State law for the processing of personal data may also provide a legal basis for further processing. In order to ascertain whether a purpose of further processing is compatible with the purpose for which the personal data are initially collected, the controller, after having met all the requirements for the lawfulness of the original processing, should take into account, inter alia:

- any link between those purposes and the purposes of the intended further processing;
- the context in which the personal data have been collected, in particular the reasonable expectations of data subjects based on their relationship with the controller as to their further use;
- the nature of the personal data;
- the consequences of the intended further processing for data subjects;
- the existence of appropriate safeguards in both the original and intended further processing operations.

Respondent Details

Please fill in the Respondent details table below:

First Name	
Last Name	
Email address	
Institution/organization/ Centre name	
Respondent Role:<br>Please indicate your role in the institution (e.g. data controller, data protection officer, chief executive officer, etc.)	

Consent:

- I hereby consent to use my contact details for the scope of the CHIEF pilot project; i.e., for being contacted in the event reviews or meetings are necessary to clarify the information provided in the questionnaire:
- **YES**
- **NO**

Signature:

Registry/HIS Profile

Questions For Analysis	Yes	No
Is the registry/HIS held in an academic organization, including academic healthcare organizations such as research hospitals, academic regional health authorities, and their research institutes?		
Does the registry participate in collaborative research with other registries (also internationally) in such frameworks as registry networks?		
Is the information contained in the registry/HIS used for research? E.g., it is used to determine the scope and design of research activities, target measurements (clinical parameters, etc.), endpoints and analytical models.		
Is the information contained in the registry/HIS used for quality monitoring? E.g., it is used for benchmarking and quality improvement in collaboration with relevant stakeholders (e.g., doctors, nurses, patients, etc.).		
Is the information contained in the registry/HIS used for policy and governance? E.g., results are used for policy (e.g., cost assessment/restructuring of services, evaluation of managers, etc.)		
Is the registry/HIS population-based?		
Does the registry/HIS have a national geographical coverage, covering more than 10% of the target population?		
Does the registry/HIS rely predominantly on provider-based datasets?		
Is the data collection continuous or periodic?		
Does the registry/HIS use administrative data?		
What are the mechanisms for data collection?		
• Electronic health records, mHealth solution, etc.		
• Data linkage		
• Analysis and reporting mechanisms (e.g., web applications, surveys, etc.)		
Are public reports produced with registry/HIS results/information?		
Have more than 10 articles published in peer reviewed journals during the last 5 years?		

Section 1. Privacy/data protection requirements

Section 1.1: Legal base for data processing: National or EU Legislation

- **If your legal base is consent, please skip this section and proceed to next section**

Questions For Analysis	Yes	No	N/A	Provide Details
1.1.1 Is the processing of health-related data in the registry/HIS based on authority of law; i.e., allowed on the bases of Union or Member State law or regional law/regulation, authorization by Supervisory Authority, etc.? ?				
Which of the following legitimate purposes for data processing justify the processing of health-related data in your registry/HIS?				
1.1.2 Is the processing of **personal data** necessary:<br>• for compliance with a legal obligation to which the controller is subject; or<br>• for the performance of a task carried out in the public interest or in the exercise of official authority vested in the controller?				
1.1.3 Is the processing of **health-related data** necessary for reasons of substantial public interests [Art 9.2 (g) of the GDPR]?				
1.1.4 Is the processing of health-related data necessary for one on the following purposes [Art 9.2 (h) of the GDPR]:<br>◦ preventive or occupational medicine;<br>◦ medical diagnosis;<br>◦ the provision of health or social care or treatment; or<br>◦ the management of health or social care systems and services?<br>and it is subject to the obligation of professional secrecy under Union or Member State law?				
1.1.5 Is the processing of health-related data necessary for reasons of public interest in the area of public health [Art 9.2 (I) of the GDPR][1]:<br>• protecting against serious cross-border threats to health; or<br>• ensuring high standards of quality and safety of health care and of medicinal products or medical devices?				
1.1.6 Is the processing of health-related data necessary [Art 9.2 (J) of the GDPR]:<br>• for archiving purposes in the public interest;<br>• scientific or historical research purposes; or<br>• statistical purposes.<br>And the safeguards required by Article 89(1) of the GDPR are in place [i.e., technical and organisational measures are in place in particular in order to ensure respect for the principle of data minimisation, including (when appropriate) the use of pseudonymisation or anonymisation techniques]?				
1.1.7 Is there a sectoral legislation (such as a law on the secondary use of health and social data), that provides a further legal base for data processing in the registry/HIS, pursuant to Art 9(4) of the GDPR?				
1.1.8 Are one or more legitimate purposes [as listed in Art. 9 (2) of the GDPR] for data processing identified and clearly documented?				

Section 1.2: Legal base for data processing: Consent

Questions For Analysis	Yes	No	N/A	Provide

1 **Recital 54 of the GDPR**: The processing of special categories of personal data may be necessary for reasons of public interest in the areas of public health without consent of the data subject. Such processing should be subject to suitable and specific measures so as to protect the rights and freedoms of natural persons. In that context, 'public health' should be interpreted as defined in Regulation (EC) No 1338/2008 of the European Parliament and of the Council (11), namely all elements related to health, namely health status, including morbidity and disability, the determinants having an effect on that health status, health care needs, resources allocated to health care, the provision of, and universal access to, health care as well as health care expenditure and financing, and the causes of mortality.

				Details
1.2.1 If consent is required, can the data subject refuse to consent to the collection or use of personal data for secondary purposes, unless required by law?				
1.2.2 If consent is required, are you able to demonstrate that consent has been freely given, informed and unambiguous?				
1.2.3 If consent is required, is it given either for one or more specified purposes?				
1.2.4 If consent is required, can the data subject withdraw his/her consent at any time?				
1.2.5 If consent is required, is a broad consent (including consent given on altruistic grounds) to further uses of registry/HIS data and/or data linkage allowed for approved health studies and research?				
1.2.6 If consent is required, is a broad consent to any further (non-health related research) uses of health data and/or data linkage allowed?				

Section 1.3: Data Subjects Rights

- **If the registry/information system does not collect personal and/or special categories directly from the individual (as a primary data source of the registry/HIS), please skip this section and proceed to next.**

Questions For Analysis	Yes	No	N/A	Provide Details
1.3.1 Does the registry/HIS allow individuals/patients to access his/her personal information, unless access is not envisaged by law?<br>For example, can individuals/patients request (including online) access to and obtain a copy of their health data ?				
1.3.2 Can individuals/patients request the rectification or erasure of personal information processed in the registry/HIS?<br>In particular, are procedures/means set up to handle data subjects requests of rectification or erasure when one of the following cases occurs?<br>• when the personal data are no longer necessary;<br>• when a data subject has withdrawn his or her consent or objects to the processing of personal data concerning him or her;<br>• when the processing of his or her personal data does not otherwise comply with the GDPR or MS law.				
1.3.3 Can individuals/patients object to the processing of his/her personal data in the registry/HIS (unless the processing is authorised by law for public interests such as public health, health threats, etc.)?				
1.3.4 Does the registry/HIS allows patients, through functionalities/on line requests, to manage the conditions for data sharing and the secondary use of their data through opt in or opt out solutions/buttons?				
1.3.5 Is the registry/HIS designed to ensure that an individual can exercise his/her right to data portability (when the processing is based on consent)?<br>Please respond N/A if the processing is not based on consent				
1.3.6 Does the registry/HIS data controller provide information and communications to individuals/patients (e.g. on the purpose of the processing, categories of data, recipients, storage duration, etc.)?				
1.3.7 Is the data subject informed of his/her right to lodge a complaint?				

Section 1.4 Accountability

Questions For Analysis	Yes	No	N/A	Provide Details
1.4.1 Have the data controller(s) and data processor(s) of the registry/HIS been nominated/established/identified?				
1.4.2 Does the data controller take records of/documents the processing activities?				
1.4.3 Do the records of processing activities contain all of the below information:<br>• the controller details<br>• the purposes of the processing<br>• a description of the categories of data subjects and of personal data<br>• the categories of recipients of the data, if any<br>• details of transfers to a third country or an international organisation, including the documentation of suitable safeguards, if any<br>• the retention periods<br>• description of the technical and organisational security measures				
1.4.4 Is a Data Protection Officer (DPO) nominated?<br>A DPO must be designated when:<br>• the processing is carried out by a public authority or body;<br>• the core activities of the controller or the processor consist of processing operations that require regular and systematic monitoring of data subjects on a large scale;<br>• the core activities of the controller or the processor consist of processing on a large scale of special categories of data.<br>If none of the above options occur, please indicate N/A				
1.4.5 Has a Data Protection Impact Assessment (DPIA) been carried out? A DPIA must be carried out when:<br>• the processing involves new technologies that could result in high risk<br>• the processing involves the processing on a large scale of special categories or "sensitive" of data<br>• the processing involves matching or combining datasets.<br>If a DPIA is not required, please indicate N/A				
1.4.6 Does the DPIA contain the minimum requirements listed in Art. 35 of the GDPR? The DPIA should include at least the following:<br>1. a systematic description of processing operations and purposes of the processing<br>2. an assessment of the necessity and proportionality of the processing operations in relation to the purposes<br>3. an assessment of the risks to the rights and freedoms of data subjects<br>4. the measures envisaged to address the risks, including safeguards, security measures and mechanisms to ensure the protection of personal data and to demonstrate compliance with data protection legislation.<br>If a DPIA is not required, please indicate N/A				
1.4.7 Are audits and checks regularly carried out to ensure compliance with accountability requirements?				

Section 1.5. Safeguarding Personal data

Section 1.5.1 Security measures for privacy protection

Questions For Analysis	Yes	No	N/A	Provide Details
1.5.1.1 Are security measures implemented to protect personal data against unauthorised or unlawful processing and against accidental loss, destruction or damage?				
1.5.1.2 In order to select the appropriate and proportioned (to the risk at stake) security measures to be implemented in the registry/HIS:<br>• Do you assess the severity and likelihood of the risks for data subjects through e.g., a DPIA or other risk assessment methods and<br>• Determine, prior to starting a processing activity, whether the purpose of processing can be achieved by using pseudonymised or anonymised data?				
1.5.1.3 Do security measures include:				
• The pseudonymisation and encryption of personal data;				
• Measures (e.g., Information Security Management System) that have the ability to ensure the ongoing confidentiality, integrity, availability and resilience of processing systems and services;				
• Measures (e.g., Information Security Management System) that have the ability to restore the availability and access to personal data in a timely manner in the event of a physical or technical incident;				
• Regular testing, assessment and evaluation of the effectiveness of the technical and organisational measures?				
1.5.1.4 Are security measures compliant with international standard according to the state of the art? For example, are one or more of the following ones used?<br>- ISO 27001 information security standard[2]<br>- ISO standards related to 27001 (such as ISO 9001, ISO 22301 and others)<br>(Please note the list is not exhaustive. Please respond "YES" and provide details if comparable standards are complied with?)				
1.5.1.5 Is compliance with international standards listed above certified by accredited registration bodies (e.g. assessment and registration bodies, certification/ registration bodies or registrars)?*				
1.5.1.6 Are employees who have permanent or regular access to personal data appropriately trained in the requirements for protecting personal information and are they aware of the relevant policies regarding breaches of security, integrity or confidentiality?				
1.5.1.7 Are one of the following plans in use in the registry/HIS to respond to incidents such as cyberattacks, disruption and crises of any kind?<br>• Incident Response Plans, which aims to contain and manage cybersecurity incidents, such as cyberattacks, and minimize their fallout;<br>• Business Continuity Plans, which aims to ensure critical operations carry on during disruptions of any kind, whether unforeseen or planned;<br>• Disaster Recovery Plans, which aims to restore IT functionality as quickly as possible after a crisis of any kind, whether a natural disaster, technological outage or cyberattack;<br>• Any other documented procedure to identify and respond to security breaches, disclosures of personal information in error, cyberattacks, etc.				

Section 1.5.2 Privacy-by-Design & Privacy-by-Default

2 ISO/IEC 27001 provides normative requirements for the development and operation of an ISMS, including a set of controls for the control and mitigation of the risks associated with the information assets which the organization seeks to protect by operating its ISMS. An organization operating an ISMS may have its conformity audited and certified. In some countries, the bodies that audit and certify conformity to specified standards are called "certification bodies", while in others they are commonly referred to as "registration bodies", "assessment and registration bodies", "certification/ registration bodies", and sometimes "registrars".

Questions For Analysis	Yes	No	N/A	Provide Details
1.5.2.1 Is privacy by Design implemented; i.e., are privacy protective measures implemented since the early stage of development of the registry/HIS and kept regularly monitored, reviewed and updated?				
1.5.2.2 Are the following organisational measures implemented by design, as appropriate?<br>• Data are pseudonymised or anonymised as soon as possible;<br>• Standards, best practices and codes of conduct are used for determining appropriate measures;<br>• Employees are trained about basic "cyber hygiene";<br>• Privacy policy is adopted.<br>Please answer yes if at least 2 out of the 4 measures are implemented.				
1.5.2.3 Are the following technical measures implemented by design, as appropriate?<br>• Pseudonymisation<br>• Anonymisation<br>• Encryption<br>• Malware detection systems<br>• Privacy and information security management systems<br>Please answer yes if at least 2 out of the 4 measures are implemented.				
1.5.2.4 Is Privacy by Default implemented; i.e., are privacy protective measures implemented by default to ensure that only personal data that are necessary for the specific purposes are processed?				
1.5.2.5 Do privacy by default measures include the following organisational measures:<br>• Prior identification of the data relevant and necessary for the purposes of the processing (data minimisation principle);<br>• Identification of the purposes of the processing to limit the extent of the processing to what is necessary for the defined purposes (purpose specification principle);<br>• Definition of the minimum retention period (data should be kept for as long as necessary);<br>• Set up of strict rules to access data to prevent unauthorised access (e.g., limiting access to only necessary and trained staff, bound by professional secrecy and confidentiality obligations).<br>Please answer yes if at least 2 out of the 4 measures are implemented.				
1.5.2.6 Does privacy by default measures include the following technical measures?<br>• Software applications, computer programs or devices that have a default setting (through choosing pre-existing or preselected values of a configurable setting) that minimise data collection and use (i.e., that limits the amount of personal data collected to what is necessary for the specific purposes);<br>• Software applications, computer programs or devices for automatic periodic data deletion;<br>Please answer yes if at least 1 out of the 2 measures are implemented.				

Section 1.5.3 Data Breach Notification and Communication

Questions For Analysis	Yes	No	N/A	Provide Details

1.5.3.1 Is the data controller mandated (within 72 hours and anyway without undue delay) to notify personal data breaches to the competent supervisory authority when they are likely to result in a risk to the rights and freedoms of data subjects)?				
1.5.3.2 Does the notification to the supervisory authority include the following information?<br>• A description of the nature of the personal data breach including where possible, the categories and approximate number of data subjects concerned and the categories and approximate number of personal data records concerned;<br>• The name and contact details of the data protection officer or other contact point where more information can be obtained;<br>• A description of the likely consequences of the personal data breach;<br>• A description of the measures taken or proposed to be taken by the controller to address the personal data breach, including, where appropriate, measures to mitigate its possible adverse effects.				
1.5.3.3 Is the data controller mandated to communicate the personal data breach to the data subject, if the data breach is likely to result in a high risk to the rights and freedoms of data subjects[3]?				
1.5.3.4 Does the communication to the data subject include the following information?<br>• The name and contact details of the data protection officer or other contact point where more information can be obtained;<br>• A description of the likely consequences of the personal data breach;<br>• A description of the measures taken or proposed to be taken by the controller to address the personal data breach, including, where appropriate, measures to mitigate its possible adverse effects.				

3 The communication to the data subject referred to in paragraph 1 (**Article 34 GDPR)** shall not be required if any of the following conditions are met: 1) the controller has implemented appropriate technical and organisational protection measures, and those measures were applied to the personal data affected by the personal data breach, in particular those that render the personal data unintelligible to any person who is not authorised to access it, such as encryption; 2) the controller has taken subsequent measures which ensure that the high risk to the rights and freedoms of data subjects is no longer likely to materialise; 3) it would involve disproportionate effort. In such a case, there shall instead be a public communication or similar measure whereby the data subjects are informed in an equally effective manner.

Section 1.5.4 Data Protection Engineering: Anonymisation

- **If you do not carry out data anonymisation please proceed to next section**

Questions For Analysis	Yes	No	N/A	Provide Details
1.5.4.1 When anonymisation is required for the further processing of personal data contained in the registry/HIS, does your centre/institution has to apply a pre-defined anonymisation process?				
1.5.4.2 Is the anonymisation process performed in compliance with the Data Protection Principles; for instance, performed confidentially, providing information to patients about the processing operation, applying security measures for data processing, storage, retention, etc.?				
1.5.4.3 Are the applied anonymisation techniques compliant with international standards (e.g., ISO/IEC 27559:2022) and continuously updated according to the state of the art?				
1.5.4.4 Is the anonymisation process documented, including the identification of the anonymisation in data life cycle stages (i.e., in data entry, collection, processing and use)?				
1.5.4.5 Are Randomization techniques (e.g., noise addition, permutation, differential privacy) used in the anonymisation process?				
1.5.4.6 Are Generalization techniques (e.g. Aggregation and K-anonymity, L-diversity/T-closeness) used in the anonymisation process?				
1.5.4.7 Is a combination of Randomization and Generalization techniques used in the anonymisation process, when suitable?				
1.5.4.8 Are additional privacy enhancing technologies used when a residual risk of re-identification could still materialise?				

Section 1.5.5 Data protection engineering: pseudonymisation

- **If you do not carry out data pseudonymisation please proceed to next section**

Questions For Analysis	Yes	No	N/A	Provide Details
1.5.5.1 When pseudonymisation is required for the further processing of personal data contained in the registry/HIS, does your centre/institution follows a standard procedure to perform data pseudonymisation ?				
1.5.5.2 Is the applied procedure compliant with international technical standards and continuously updated according to the state of the art?				
1.5.5.3 Does pseudonymisation involve the followings?<br>• Personal data can no longer be attributed to a specific data subject without the use of additional information (re-identification key) and<br>• Such additional information is kept separately and subject to appropriate technical and organizational measures.				
1.5.5.4 Do you select the appropriate pseudonymisation technique(s):<br>• case by case, considering the type of data to be processed; and<br>• by considering the pseudonymisation policy that will be implemented?				
1.5.5.5 Does your centre/institution use one or more of the following techniques, as suitable?<br>• Counter: Monotonic counter which starts at a certain value and is increased each time a new pseudonym is necessary<br>• Random number: Random value extracted between a minimum and a maximum boundary each time a new pseudonym is necessary<br>• Hash function: One-way (non-reversible) cryptographic function transforming input personal data in fixed-length values<br>• Hash-based message authentication code (HMAC): One-way (non-reversible) cryptographic function adding a key that makes it less predictable than a hash function<br>• Encryption: Two-way (reversible) cryptographic function transforming an input personal data in values that can be re-transformed in its original format using a key				
1.5.5.6 Does your centre/institution implement one of the following pseudonymisation policies, as suitable:<br>• Deterministic pseudonymisation – always using the same pseudonym for the same data;<br>• Document randomised pseudonymisation – using the same pseudonym for the same data only within a consistent scope;<br>• Fully randomised pseudonymisation – always using a different pseudonym for the same data.				

Section 1.6: Data Linkage

- **If you do not perform data linkage please proceed to next section/sub-section**

Questions For Analysis	Yes	No	N/A	Provide Details
1.6.1 Do you perform data linkages operations in the registry/HIS?				
1.6.2 Are unique personal identifiers (e.g., social insurance/security numbers) used for the purposes of linking across multiple databases (not de-identified or pseudonymised)?				
1.6.3 Is record linkage performed using identifying attributes (e.g., name, sex, birth date, address) from the registry/HIS or other health records?				
1.6.4 Do you apply standard practices for deleting direct identifiers such as names and personal identifiers (e.g. creating pseudonyms from direct identifiers) for the performance of data linkages?				
1.6.5 Is the de-identification and/or pseudonymisation methodology documented?				
1.6.6 Do you use a pre-defined process for the assessment of the risk of data re-identification?				
1.6.7 Do you use additional protective practices for the treatment of attributes that pose a higher re-identification risk (such as rare diseases, exact dates, locations, or ethnic origins)?				
1.6.8 In order **to estimate reliable health indicators,** do you consider and assess the data linkage limitations with regard to:<br>• Research question and study design;<br>• Original data quality and data lineage;<br>• Data context knowledge;<br>• Data linkage capacities?				
1.6.9 In order **to estimate reliable health indicators, d**o you continuously build data linkage capacities through:<br>• Involvement of data domain experts;<br>• Use of state-of-the-art technical linkage approaches and methodologies;<br>• Implementing comprehensive data governance with a focus on all data quality dimensions;<br>○ Data transparency through comprehensive catalogues;<br>○ Data availability through various services and access technologies, etc.				
1.6.10 In order **to estimate reliable health indicators, d**o you select the most appropriate technical methodologies for data linkage leveraging from multidisciplinary team of experts in social sciences, medical experts in the research domain, regulatory and decision-makers, as well as analytical and design experts?				

Section 2. Data Governance

Section 2.1 Governance Framework

Questions For Analysis	Yes	No	N/A	Provide Details
2.1.1 Has your centre/institution established a data governance framework?				
2.1.2 Does your centre/institution have clearly defined roles and responsibilities for key stakeholders in data governance, including data stewards, data processors, data managers, and other relevant roles such as data protection officers and IT teams?				
2.1.3 Has your centre/institution established formal policies and procedures governing the entire data lifecycle, including data collection, processing, storage, security, and disposal?				
2.1.4 Are these policies regularly reviewed and updated to ensure alignment with international standards, regulatory requirements, and best practices?				
2.1.5 Does your institution have a comprehensive and documented process for identifying, assessing, and mitigating cybersecurity risks?				
2.1.6 Does this process include regular risk assessments, vulnerability testing, incident response planning, and the implementation of measures to address emerging threats and ensure compliance with relevant cybersecurity standards?				
2.1.7 Does your institution have a documented process for implementing data protection engineering techniques, including data minimization, pseudonymisation, encryption, and other methods to ensure data security and compliance with privacy regulations?				

Section 2.2 Access Control and Auditing

Questions For Analysis	Yes	No	N/A	Provide Details
2.2.1 Does your institution implement secure authentication mechanisms (e.g., multi-factor authentication, strong password policies) to control access to sensitive data?				
2.2.2 Are access permissions managed through a role-based access control (RBAC) system to ensure users only have access to the data necessary for their role?				
2.2.3 Are access rights to sensitive data reviewed and updated periodically to ensure ongoing compliance with authorisation policies?				
2.2.4 Is there a procedure in place to immediately revoke access for users who leave the organisation or change roles?				
2.2.5 Are third-party collaborators or contractors required to comply with your institution's authentication and authorisation protocols?				
2.2.6 Does your institution maintain audit trails to log all access to and modifications of sensitive data?				
2.2.7 Are audit logs configured to track who accessed data, what changes were made, and when the actions occurred?				
2.2.8 Are mechanisms in place to alert administrators or stakeholders to any unauthorised access or modification detected through audit logs?				

Section 2.3 Data Quality and Integrity

Questions For Analysis	Yes	No	N/A	Provide Details
2.3.1 Has your institution established formal data quality assurance standards and protocols to ensure accurate and reliable data collection and management?				
2.3.2 Are these standards and protocols documented and communicated to all relevant staff involved in data collection and management?				
2.3.3 Are responsibilities for data quality assurance clearly assigned to specific roles or teams?				
2.3.4 Does your institution regularly validate and verify data to ensure its accuracy, completeness, and timeliness?				
2.3.5 Are automated tools or manual processes used for data validation and verification?				
2.3.6 Are discrepancies or errors identified during validation promptly corrected and documented?				
2.3.7 Has your institution implemented formal data cleaning procedures and processes to remove errors, inconsistencies, or irrelevant information from datasets?				
2.3.8 Are feedback mechanisms in place to address issues reported by data users or stakeholders regarding data quality?				

Section 2.4 Training and Awareness

Questions For Analysis	Yes	No	N/A	Provide Details
2.4.1 Does your centre/institution provide regular training on data governance principles and practices for staff involved in data management?				
2.4.2 If yes, Is training required on a regular basis (e.g., annually, biannually)?				
2.4.3 If, yes Are these training sessions tailored to the specific roles and responsibilities of staff?				
2.4.4 Does your institution have a mechanism to evaluate the effectiveness of training and awareness programs related to data governance?				
2.4.5 Do these training programmes include an overview of regulatory requirements (e.g., GDPR, EHDS Regulation) and best practices?				
2.4.6 Are registry participants informed about how their data is protected, stored, and used, and are they provided with clear guidelines on their rights to access, correct, or delete their data?				
2.4.7 Are registry participants provided with clear guidelines on their rights to access, correct, or delete their data?				

Section 2.5 Data Sharing and Collaboration

Questions For Analysis	Yes	No	N/A	Provide Details
2.5.1 Are data controllers allowed to share readily identifiable health data for statistics or research with national public authorities and/or academic or private organisations for non-commercial purposes?				
2.5.2 Are data controllers allowed to share de-identified or pseudonymised health data for statistics or research with national public authority and/or academic or private organisations for non-commercial purposes?				
2.5.3 Are data controllers allowed to share readily identifiable health data for statistics or research with foreign public authorities and/or academic or private organisations for non-commercial purposes (cross-border data flow)?				
2.5.4 Are data controllers allowed to share de-identified or pseudonymised health data for statistics and research with another foreign public authority and or academic or private organisations for non-commercial purposes?				
2.5.5 Do you have a standard data sharing agreement for disclosing data (or multiple standard ones for different types of data requestors)?				
2.5.6 Does your data sharing agreement require specific privacy/security practices at the data recipient's site?				
2.5.7 Does your data sharing agreement state the penalties for non-compliance with privacy or security practices (e.g., in cases of data breaches)?				
2.5.8 Does your data sharing agreement specify procedures or restrictions regarding the publication of data (indirect disclosure) and data retention requirements?				
2.5.9 Are data recipients required to provide evidence of compliance with privacy and security practices outlined in the agreement?				
2.5.10 Are mechanisms in place to monitor and audit data sharing activities to detect potential misuse or non-compliance?				

Section 2.6. Openness and Transparency

- **If you do not collect and/or process personal data, please fill in this section with all N/A and proceed to next section.**

Questions For Analysis	Yes	No	N/A	Provide Details
2.6.1 Are data subjects (e.g., individuals whose data is collected) consulted and/or informed about how their data is accessed, used, stored, and shared in the registry/HIS?				
2.6. 2 Does your institution have a formal communication plan or strategy to explain to the public how personal information is collected, managed, and protected? If NOT, please respond N/A to 2.6.3, 2.6.4, 2.6.5.				
2.6.3 Does the communication plan or strategy include details on:<br>• The **benefits** of processing personal data?<br>• The **risks** associated with processing personal data?<br>• The **risk mitigation strategies** in place to protect personal information?				
2.6.4 Does the communication plan/strategy include publicly accessible information, such as through a website, that describes the content of datasets and identifies data controllers and processors responsible for managing these datasets?				
2.6.5 Does the communication plan/strategy include publicly accessible information, such as through a website, that describes applications for approval of the health datasets processing, including dataset linkages, as well as approval decisions for such applications?				
2.6.6 Is a certification/accreditation process for the processing of health data for research and statistics implemented?				
2.6.7 Does your institution have a process in place to **respond to inquiries or complaints** about data handling and ensure transparency in addressing concerns?				

Section 2.7 Health Research Projects Approval Process

Questions For Analysis	Yes	No	N/A	Provide Details
2.7.1 When research projects are conducted using health-related data from the registry or health information system (HIS), is there a national, regional, or local project approval body that authorizes such research?				
2.7.2 Are the project approval bodies multidisciplinary, involving relevant stakeholders with no vested interest in the approval process (legal experts, privacy experts, statistical experts, patients and researchers)?				
2.7.3 Are the approval bodies publicly identified, with transparency regarding their role, responsibilities, and membership composition?				
2.7.4 Are the criteria that the body follows for project approval publicly identified/accessible, including timeliness of approval decisions?				
2.7.5 Are approval bodies accountable for the timeliness and quality of their services?				
2.7.6 Are there clear and accessible complaint or appeal procedures to challenge or contest the decisions made by the approval bodies?				

Section 3. Interoperability & Preparedness to the requirements of the EHDS Regulation

Section 3.1 Electronic Health Records (EHRs) content requirements and the European Electronic Health Data Exchange Format

- **If the registry/HIS does not communicate with an EHR system, please skip this section and proceed to next.**

Questions For Analysis	Yes	No	N/A	Provide Details
3.1.1 Are EHRs a routine data source of the registry/HIS?				
3.1.2 Does the EHR include a patient summary?				
3.1.3 Does the patient summary includes the following content?				
1. Personal details				
2. Contact information				
3. Information on insurance				
4. Allergies				
5. Medical alerts				
6. Vaccination/prophylaxis information, possibly in the form of a vaccination card				
7. Current, resolved, closed or inactive problems				
8. Textual information related to medical history				
9. Medical devices and implants				
10. Procedures				
11. Functional status				
12. Current and relevant past medicines				
13. Social history observations related to health				
14. Pregnancy history				
15. Patient provided data				
16. Observation results pertaining to the health condition				
17. Plan of care				
18. Information on a rare disease e.g. details about impact/characteristics of disease				
3.1.4 Does the EHR also includes:				
• eprescriptions/dispensations				
• laboratory results				
• medical imaging and reports				
• hospital discharge reports				
3.1.5 Does the EHR system adopt the European Electronic Health Data Exchange Format envisaged in the 2019 Commission Recommendation[11]?				
(a) Does the EHR system adopts the following content representations for Patient Summary and ePrescription/eDispensation:<br>○ Health Level Seven (HL7) Clinical Document Architecture (CDA) Release 2<br>○ Level 3 and Level 1 (PDF (3)/A); or<br>○ CEN/ISO 27269:2021 Health informatics Int.Patient Summary standard?				
(b) Does the EHR system adopt the following content representations for laboratory results, medical imaging reports and hospital discharge reports:<br>○ Health Level Seven (HL7) Clinical Document Architecture (CDA) Release 2 Level 3 or Level 1 (PDF (1)/A).				
(c) Does the EHR system adopt the following content representations for medical imaging:<br>○ Digital Imaging and Communications in Medicine (DICOM)				

Section 3.2 **Registries/HIS requirements for interoperability with EHR Systems' (EHDS Regulation)**

- **If the registry/HIS does not communicate with the EHR system, please skip this section and proceed to next.**

Questions For Analysis	Yes	No	N/A	Provide Details
3.2.1 Does the registry/HIS allow personal electronic health data to be shared between health professionals or other entities from the health system (e.g., the EHR system), and between health professionals and patient or health professional portals?				
3.2.2 Do data controllers/data holders of disease registries/HIS ensure that health information is collected/recorded according to harmonised data formats and coding systems to allow interoperability with the EHR system?				
3.2.3 Does the registry/HIS fulfil the general, interoperability and security requirements envisaged for EHR systems by the EHDS Regulation, including the use of the European Electronic Health Record Exchange Format?				
3.2.4 Does the registry/HIS allow the exercise (by data subjects) of the right to access, rectification, restriction, portability and to object (with regard to the priority categories of personal electronic health data for primary use indicated in Art. 5 of the EHDS Regulation[4]), via an electronic health data access service that provides for an immediate answer (e.g., data are directly downloadable from the electronic health data access service/patient portal)?				
3.2.5 Does the registry/HIS allow data subjects to transmit their electronic health data, including inferred data, in the European electronic health record exchange format, irrespective of the legal basis for processing the electronic health data?				
3.2.6 Do data controllers have an obligation to ensure data portability?				

4 The priority categories of personal electronic health data for primary use indicated in Art. 5 of the EHDS Regulation are: patient summaries; electronic prescriptions; electronic dispensations; medical imaging studies and related imaging reports; medical test results, including laboratory and other diagnostic results and related reports; discharge reports.

Section 3.3 Data Documentation, Data Quality and Utility Label (EHDS Regulation)

Questions For Analysis	Yes	No	N/A	Provide Details
3.3.1 Does the registry/HIS provide for the Datasets Description?				
3.3.2 Does the dataset description allows the following?<br>• Data controllers are able to provide a metadata catalogue of the datasets they hold;<br>• Datasets include information concerning the source, the scope, the main characteristics and nature of electronic health data contained in the health dataset.				
3.3.3 Does electronic health data collected and processed comply with Data Quality and Utility Standards?				
3.3.4 Are Data Quality and Utility Standards ensured in each of the following elements?				
1. data documentation: meta-data, support documentation, data model, data dictionary, standards used, provenance;				
2. technical quality, showing the completeness, uniqueness, accuracy, validity, timeliness and consistency of the data;				
3. data quality management processes: level of maturity of the data quality management processes, including review and audit processes, biases examination;				
4. coverage: representation of multi-disciplinary electronic health data, representativity of population sampled, average time-frame in which a natural person appears in a dataset;				
5. information on access and provision: time between the collection of the electronic health data and their addition to the dataset, time to provide electronic health data following electronic health data access application approval;				
6. information on data enrichments: merging and adding data to an existing dataset, including links with other datasets.				

Section 3.4 Secondary use of health data (EHDS Regulation)

Questions For Analysis	Yes	No	N/A	Provide Details
3.4.1 Are data controllers of the registry/HIS allowed to use data for the following secondary purposes?				
1. public interest in the area of public and occupational health, such as protection against serious cross-border threats to health, public health surveillance or ensuring high levels of quality and safety of healthcare and of medicinal products or medical devices;				
2. to support public sector bodies or Union institutions, agencies and bodies including regulatory authorities, in the health or care sector to carry out their tasks defined in their mandates;				
3. to produce national, multi-national and EU level official statistics related to health/care sectors;				
4. education or teaching activities in health or care sectors;				
5. scientific research related to health or care sectors;				
6. development and innovation activities for products or services contributing to public health or social security, or ensuring high levels of quality and safety of health care, of medicinal products or of medical devices;				
7. training, testing and evaluating of algorithms, including in medical devices, AI systems and digital health applications, contributing to the public health or social security, or ensuring high levels of quality and safety of health care, of medicinal products or of medical devices				
8. providing personalised healthcare consisting in assessing, maintaining or restoring the state of health of natural persons, based on the health data of other natural persons.				
3.4.2 When using data for secondary purposes, do you check if they are compatible with the purposes for which data were previously collected, unless the processing is required by law?				
3.4.3 If personal data is to be processed for a secondary purpose not previously identified, is consent required (unless the processing is authorised by law, Regulation, or involves a disproportioned effort.)?				
3.4.4 Is data pseudonymised before it is used for any secondary purpose, including data linkage?				
3.4.5 Is information anonymised when used for planning, management and/or evaluation purposes?				
3.4.6 Are the following categories of electronic data available for secondary use?				
• electronic health data from EHRs;				
• data on factors impacting on health, including socio-economic, environmental and behavioural determinants of health				
• aggregated data on healthcare needs, resources allocated to healthcare, the provision of and access to healthcare, healthcare expenditure and financing				
• Pathogen data, impacting on human health				
• health-related administrative data, including dispensation, claims and reimbursement data				
• human genetic, epigenomic and genomic data				
• other human molecular data e.g. proteomic transcriptomic, metabolomic, lipidomic and other omics data				
• Automatically generated personal electronic health data, through medical devices				
• data from wellness applications				
• data on professional status, specialisation and institution of health professionals involved in the treatment of a natural person				
• Population-based health data registries (public health registries)				
• data from medical registries and mortality registries				
• data from clinical trials, clinical studies and clinical investigations subject to Regulation (EU) 536/2014, Regulation [SOHO], Regulation (EU) 2017/745 and Regulation (EU) 2017/746, respectively				
• other health data from medical devices				
• data from registries for medicinal products and medical devices				
• data from research cohorts, questionnaires and surveys related to health, after the first publication of results				
• electronic health data from biobanks and dedicated databases				

Section 4. Ethics: Compliance with Ethics Principles on Data Protection in Research Projects

- **If you do not carry out research projects with the registry/HIS data, please skip this section.**

Questions For Analysis	Yes	No	N/A	Provide Details
4.1 Do you conduct regional/national/European/international research projects using health related data contained in the registry/HIS?				
4.2 Is a Data Protection Officer (DPO) nominated (or contacted if a DPO has been already appointed by your institution) and his/her contact details made available to all data subjects involved in the research?<br>In case you are not required to appoint a DPO under the GDPR, is a detailed data protection policy for the project made available to data subjects?				
4.3 Are detailed information on the informed consent procedures with regard to data processing, including templates of the informed consent forms and information sheets provided in research protocols and stored/kept on file for the duration of the project or as required by law?				
4.4 Does the research protocol include a sound and detailed justification for the processing of health-related data?				
4.5 Does the research protocol explain how all of the data you intend to process is relevant and limited to the purposes of the research project (in accordance with the ‘data minimisation ‘principle)?				
4.6 Have you evaluated the ethics risks related to the data processing activities of the project, including also an opinion (from the DPO) if data protection impact assessment should be conducted under art.35 General Data Protection Regulation 2016/679?				
4.7 Does the research protocol include a description of the technical and organisational measures that will be implemented to safeguard the rights and freedoms of the data subjects/research participants?				
4.8. Does the research protocol include a description of the security measures, including anonymisation/pseudonymisation techniques, implemented to prevent unauthorised access to personal data or disclosure in error, as well as the monitoring, review and auditing processes?				
4.9 In case the research project involves personal data transfers from the EU to a non-EU country or international organisation, have you ensured that such transfers are in accordance with Chapter V of the General Data Protection Regulation 2016/679?				
4.10 In case the research project involves personal data transfers from a non-EU country to the EU (or another third state), have you ensured that such transfers comply with the laws of the country in which the data was collected?				
4.11 In case the research project involves the further processing of previously collected personal data, have you ensured and able to demonstrate that a lawful basis for the data processing and that the appropriate technical and organisational measures are in place to safeguard the rights of the data subjects?				
4.12 Is ethics approval from the competent Ethics Review Board or Authorization from the competent Data Protection Authority sought, unless this requirement is waived by law?				

2. DIGA Scoring system

Section 1. Privacy/data protection requirements

Section 1.1: Legal base for data processing: National or EU Legislation

Questions For Analysis	Yes	No	N/A	Scoring
1.1.1	3	0	0	• Max score = 7<br>• SUM of YES from 1.1.2 to 1.1.7 = 3<br>• YES to one of the questions from 1.1.2 to 1.1.7 = 3
1.1.2	1	0	0	
1.1.3	1	0	0	
1.1.4	1	0	0	
1.1.5	1	0	0	
1.1.6	1	0	0	
1.1.7	1	0	0	
1.1.8	1	0	0	

Section 1.2: Legal base for data processing: Consent

Questions For Analysis	Yes	No	N/A	Scoring
1.2.1	1	0	0	• Max score =6
1.2.2	1	0	0	
1.2.3	1	0	0	
1.2.4	1	0	0	
1.2.5	1	0	0	
1.2.6	0	1	0	

Section 1.3: Data Subjects Rights

Questions For Analysis	Yes	No	N/A	Scoring
1.3.1	1	0	0	• Max score= 7
1.3.2	1	0	0	
1.3.3	1	0	0	
1.3.4	1	0	0	
1.3.5	1	0	1	
1.3.6	1	0	0	
1.3.7	1	0	0	

Section 1.4 Accountability

Questions For Analysis	Yes	No	N/A	Scoring
1.4.1	1	0	0	• **Max score=7**
1.4.2	1	0	0	
1.4.3	1	0	0	

1.4.4	1	0	1	
1.4.5	1	0	1	
1.4.6	1	0	1	
1.4.7	1	0	0	

Section 1.5. Safeguarding Personal data

Section 1.5.1 Security measures for privacy protection

Questions For Analysis	Yes	No	N/A	Scoring
1.5.1.1	1	0	0	• Max score: 10
1.5.1.2	1	0	0	
1.5.1.3.1	1	0	0	
1.5.1.3.2	1	0	0	
1.5.1.3.3	1	0	0	
1.5.1.3.4	1	0	0	
1.5.1.4	1	0	0	
1.5.1.5	1	0	0	
1.5.1.6	1	0	0	
1.5.1.7	1	0	0	

Section 1.5.2 Privacy-by-Design & Privacy-by-Default

Questions For Analysis	Yes	No	N/A	Scoring
1.5.2.1	1	0	0	• Max score=6
1.5.2.2	1	0	0	
1.5.2.3	1	0	0	
1.5.2.4	1	0	0	
1.5.2.5	1	0	0	
1.5.2.6	1	0	0	

Section 1.5.3 Data Breach Notification and Communication

Questions For Analysis	Yes	No	N/A	Scoring
1.5.3.1	1	0	0	• Max score= 4
1.5.3.2	1	0	0	
1.5.3.3	1	0	0	
1.5.3.4	1	0	0	

Section 1.5.4 Data Protection Engineering: Anonymisation

Questions For Analysis	Yes	No	N/A	Scoring
1.5.4.1	1	0	0	• Max score=7
1.5.4.2	1	0	0	
1.5.4.3	1	0	0	

1.5.4.4 I	1	0	0	• If 1.5.4.5 = Yes and 1.5.4.6 = NO; then 1.5.4.5 = 1 and 1.5.4.6 = 1<br>• If 1.5.4.5 = NO and 1.5.4.6 = YES; then, 1.5.4.5 = 1 and 1.5.4.6 = 1
1.5.4.5	1	0	0	
1.5.4.6	1	0	0	
1.5.4.7	0	0	0	
1.5.4.8	1	0	0	

Section 1.5.5 Data Protection Engineering: Pseudonymisation

Questions For Analysis	Yes	No	N/A	Scoring
1.5.5.1	1	0	0	• Max score=6
1.5.5.2	1	0	0	
1.5.5.3	1	0	0	
1.5.5.4	1	0	0	
1.5.5.5	1	0	0	
1.5.5.6	1	0	0	

Section 1.6: Data Linkage

Questions For Analysis	Yes	No	N/A	Scoring
1.6.1	1	0	0	• Max score = 10
1.6.2	1	0	0	
1.6.3	0	1	0	
1.6.4	1	0	0	
1.6.5	1	0	0	
1.6.6	1	0	0	
1.6.7	1	0	0	
1.6.8	1	0	0	
1.6.9	1	0	0	
1.6.10	1	0	0	

Section 2. Data Governance

Section 2.1 Governance Framework

Questions For Analysis	Yes	No	N/A	Scoring
2.1.1	1	0	0	• Max score: 7
2.1.2	1	0	0	
2.1.3	1	0	0	
2.1.4	1	0	0	
2.1.5	1	0	0	
2.1.6	1	0	0	
2.1.7	1	0	0	

Section 2.2 Access Control and Auditing

Questions For Analysis	Yes	No	N/A	Scoring
2.2.1	1	0	0	• Max score= 8
2.2.2	1	0	0	
2.2.3	1	0	0	
2.2.4	1	0	0	
2.2.5	1	0	0	
2.2.6	1	0	0	
2.2.7	1	0	0	
2.2.8	1	0	0	

Section 2.3 Data Quality and Integrity

Questions For Analysis	Yes	No	N/A	Scoring
2.3.1	1	0	0	• Max score= 8
2.3.2	1	0	0	
2.3.3	1	0	0	
2.3.4	1	0	0	
2.3.5	1	0	0	
2.3.6	1	0	0	
2.3.7	1	0	0	
2.3.8	1	0	0	

Section 2.4 Training and Awareness

Questions For Analysis	Yes	No	N/A	Scoring
2.4.1	1	0	0	• Max score= 7
2.4.2	1	0	0	
2.4.3	1	0	0	
2.4.4	1	0	0	
2.4.5	1	0	0	
2.4.6	1	0	0	
2.4.7	1	0	0	

Section 2.5 Data Sharing and Collaboration

Questions For Analysis	Yes	No	N/A	Scoring
2.5.1	0	1	0	• Max score= 10
2.5.2	1	0	0	
2.5.3	0	1	0	
2.5.4	1	0	0	
2.5.5	1	0	0	
2.5.6	1	0	0	
2.5.7	1	0	0	
2.5.8	1	0	0	
2.5.9	1	0	0	
2.5.10	1	0	0	

Section 2.6. Openness and Transparency

Questions For Analysis	Yes	No	N/A	Scoring
2.6.1	1	0	0	• Max score = 7
2.6. 2	1	0	0	
2.6.3	1	0	0	
2.6.4	1	0	0	
2.6.5	1	0	0	
2.6.6	1	0	0	
2.6.7	1	0	0	

Section 2.7 Health Research Projects Approval Process

Questions For Analysis	Yes	No	N/A	Scoring
2.7.1	1	0	0	• Max score= 6
2.7.2	1	0	0	
2.7.3	1	0	0	
2.7.4	1	0	0	
2.7.5	1	0	0	
2.7.6	1	0	0	

Section 3. Preparedness to the requirements of the EHDS Regulation

Section 3.1 Electronic Health Records (EHRs) content requirements and the European Electronic Health Data Exchange Format

Questions For Analysis	Yes	No	N/A	Scoring
3.1.1	1	0	0	• Max score of the factor =11<br>• Question 6.1.3.1-18 Max score = 2:<br>○ If the number of YES <=9, score = 1<br>○ If the number of YES = from 10 to 18, score = 2
3.1.2	1	0	0	
3.1.3.1	1	0	0	
3.1.3.2	1	0	0	
3.1.3.3	1	0	0	
3.1.3.4	1	0	0	
3.1.3.5	1	0	0	
3.1.3.6	1	0	0	
3.1.3.7	1	0	0	
3.1.3.8	1	0	0	
3.1.3.9	1	0	0	
3.1.3.10	1	0	0	
3.1.3.11	1	0	0	
3.1.3.12	1	0	0	
3.1.3.13	1	0	0	
3.1.3.14	1	0	0	
3.1.3.15	1	0	0	
3.1.3.16	1	0	0	
3.1.3.17	1	0	0	
3.1.3.18	1	0	0	
3.1.4.1	1	0	0	
3.1.4.2	1	0	0	
3.1.4.3	1	0	0	
3.1.4.4	1	0	0	
3.1.5.1	1	0	0	
3.1.5.2	1	0	0	
3.1.5.3	1	0	0	

Section 3.2 Registries/HIS requirements for interoperability with EHR Systems' (EHDS Regulation)

Questions For Analysis	Yes	No	N/A	Scoring
3.2.1	1	0		• Max score = 6
3.2.2	1	0		
3.2.3	1	0		
3.2.4	1	0		
3.2.5	1	0		
3.2.6	1	0		

Section 3.3 Data Documentation, Data Quality and Utility Label (EHDS Regulation)

Questions For Analysis	Yes	No	N/A	Scoring
3.3.1	4	0	0	• Max score = 15<br>Max score for Q.3.3.4.1-3.3.4.6 = 3<br>• If Q.3.3.4.1-3.3.4.6 include 1 YES = 0<br>• If Q.3.3.4.1-3.3.4.6 include 2 YES = 1<br>• If Q.3.3.4.1-3.3.4.6 include from 3 to 4 YES = 2<br>• If Q.3.3.4.1-3.3.4.6 include from 5 to 6 YES = 3
3.3.2	4	0	0	
3.3.3	4	0	0	
3.3.4.1	1	0	0	
3.3.4.2	1	0	0	
3.3.4.3	1	0	0	
3.3.4.4	1	0	0	
3.3.4.5	1	0	0	
3.3.4.6	1	0	0	

Section 3.4 Secondary use of health data (EHDS Regulation)

Questions For Analysis	Yes	No	N/A	Scoring
3.4.1.1	1	0	0	• Max score = 12<br>• Max score for question 3.4.1.1-8 = 4<br>◦ Number of YES <=1 = mark of 0<br>◦ Number of YES from 2 to 4 = mark of 2<br>◦ Number of YES from 5 to 8= 4<br>• Max score for question 3.4.6.1-17 = 4<br>◦ Number of YES <=5 = mark of 0<br>◦ Number of YES from 6 to 12 = mark of 2<br>◦ Number of YES from 13 to 17 = mark of 4
3.4.1.2	1	0	0	
3.4.1.3	1	0	0	
3.4.1.4	1	0	0	
3.4.1.5	1	0	0	
3.4.1.6	1	0	0	
3.4.1.7	1	0	0	
3.4.1.8	1	0	0	
3.4.2	1	0	0	
3.4.3	1	0	0	
3.4.4	1	0	0	
3.4.5	1	0	0	
3.4.6.1	1	0	0	
3.4.6.2	1	0	0	
3.4.6.3	1	0	0	
3.4.6.4	1	0	0	
3.4.6.5	1	0	0	
3.4.6.6	1	0	0	
3.4.6.7	1	0	0	
3.4.6.8	1	0	0	
3.4.6.9	1	0	0	
3.4.6.10	1	0	0	
3.4.6.11	1	0	0	
3.4.6.12	1	0	0	
3.4.6.13	1	0	0	
3.4.6.14	1	0	0	
3.4.6.915	1	0	0	
3.4.6.16	1	0	0	
3.4.6.17	1	0	0	

Section 4. Ethics

Section 4.1 Compliance with Ethics Principles on Data Protection in Research Projects

Questions For Analysis	Yes	No	N/A	Scoring
4.1	1	0	0	• Max score = 12
4.2	1	0	0	
4.3	1	0	0	
4.4	1	0	0	
4.5	1	0	0	
4.6	1	0	0	
4.7	1	0	0	
4.8	1	0	0	
4.9	1	0	0	
4.10	1	0	0	
4.11	1	0	0	
4.12	1	0	0	
