# Supplementary material for: Data protection, interoperability and governance assessment tool: results from a proof-of-concept survey
Source: Front Digit Health. 2025 Oct 27;7:1685774. doi: 10.3389/fdgth.2025.1685774 (PMC12597924; doi:10.3389/fdgth.2025.1685774)
Supplement: Supplementary file 2 [file Datasheet2.pdf]

DIGA: USERS FEEDBACK QUESTIONNAIRE

N.	Questions	Very low	Adequate	Very good
1	How do you rank the suitability of the DIGA questionnaire as a self-assessment tool by scoring the adherence of participating centres to data protection, data governance, interoperability and ethics requirements?			
2	How do you rank the suitability of the questionnaire as a check-list to be used by data controllers/holders of disease registries/health information systems to evaluate their level of privacy protection, interoperability, adherence to data governance best practices and ethics compliance?			
3	How do you rank the ability of the DIGA questionnaire to evaluate the overall level of data protection, data governance, interoperability and ethics of the sample of registries/health information systems that are involved in the survey, considering that the reliability of the overall assessment grows with the growing of the sample?			
4	How do you rank the anonymity feature of the individual self-assessment?			
5	How do you rank the suitability of the DIGA questionnaire to evaluate the heterogeneity of the sample in the implementation of data protection, data governance, interoperability and ethics?			
6	How do you rank the suitability of the DIGA questionnaire to identify key areas of concern in the implementation of privacy protection, data governance, interoperability and ethics?			
7	How do you rank the complexity of the DIGA Questionnaire, in terms of difficulty in answering questions?			
8	How do you rank the overall utility of the DIGA tool as a quality improvement means?			
9	How do you rank the comprehensiveness of the questionnaire, considering that the sections are independent to each other and can be filled in according to areas of interests, applicability or needs of the respondents without impacting on centres' scores?			
10	Do you have any further comment, remark, suggestion for improvement of the DIGA tool, etc.	Open response:		
